# *Apo2ph4*: A Versatile Workflow for the Generation of Receptor-based Pharmacophore Models for Virtual Screening

**DOI:** 10.1021/acs.jcim.2c00814

**Published:** 2022-12-16

**Authors:** Jörg Heider, Jonas Kilian, Aleksandra Garifulina, Steffen Hering, Thierry Langer, Thomas Seidel

**Affiliations:** † Department of Pharmaceutical Sciences, University of Vienna, Josef-Holaubek-Platz 2, 1090Vienna, Austria; ‡ Vienna Doctoral School of Pharmaceutical, Nutritional and Sport Sciences, University of Vienna, Josef-Holaubek-Platz 2, 1090Vienna, Austria; § Department of Biomedical Imaging and Image-Guided Therapy, Division of Nuclear Medicine, Medical University of Vienna, Währinger Gürtel 18-20, 1090Vienna, Austria; ∥ Division of Pharmacology and Toxicology, Department of Pharmaceutical Sciences, University of Vienna, Josef-Holaubek-Platz 2, 1090Vienna, Austria

## Abstract

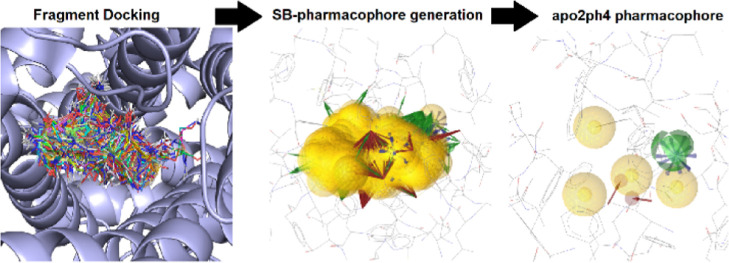

Pharmacophore models are widely used as efficient virtual screening (VS) filters for the target-directed enrichment of large compound libraries. However, the generation of pharmacophore models that have the power to discriminate between active and inactive molecules traditionally requires structural information about ligand–target complexes or at the very least knowledge of one active ligand. The fact that the discovery of the first known active ligand of a newly investigated target represents a major hurdle at the beginning of every drug discovery project underscores the need for methods that are able to derive high-quality pharmacophore models even without the prior knowledge of any active ligand structures. In this work, we introduce a novel workflow, called *apo2ph4*, that enables the rapid derivation of pharmacophore models solely from the three-dimensional structure of the target receptor. The utility of this workflow is demonstrated retrospectively for the generation of a pharmacophore model for the M2 muscarinic acetylcholine receptor. Furthermore, in order to show the general applicability of *apo2ph4*, the workflow was employed for all 15 targets of the recently published LIT-PCBA dataset. Pharmacophore-based VS runs using the *apo2ph4*-derived models achieved a significant enrichment of actives for 13 targets. In the last presented example, a pharmacophore model derived from the etomidate site of the α1β2γ2 GABA_A_ receptor was used in VS campaigns. Subsequent in vitro testing of selected hits revealed that 19 out of 20 (95%) tested compounds were able to significantly enhance GABA currents, which impressively demonstrates the applicability of *apo2ph4* for real-world drug design projects.

## Introduction

According to the official IUPAC definition, “a pharmacophore is the ensemble of steric and electronic features that is necessary to ensure the optimal supramolecular interactions with a specific biological target structure and to trigger (or to block) its biological response.”^1^ With an appropriate pharmacophore model in hand, compound libraries containing millions of compounds may be subjected to virtual screening (VS) in order to identify compounds showing the same feature pattern as the query pharmacophore.^2^ If the query pharmacophore models the interactions of compounds showing activity toward a particular target, the obtained hit lists can be expected to contain a share of likewise active molecules that is significantly higher than the one in randomly selected subsets. Hit lists enriched with active compounds represent a considerable economic advantage because testing hundreds of promising VS hits rather than testing thousands of compounds is clearly more resource saving.^3^ Due to its simplicity, pharmacophore-based VS is also computationally quite efficient and millions of compounds can be screened in relatively short amounts of time—something that is out of reach for traditional docking-based approaches.^4−6^ The generation of pharmacophore models utilizing co-crystal structures in a so-called structure-based (SB) manner is relatively straightforward and done by identifying present non-covalent interactions between a bound ligand and residues at the binding pocket surface.^7^ Several advanced tools, for example, LigandScout,^8^ Phase,^9^ or Catalyst,^9^ are available for this purpose. In cases where structural information about a ligand–target complex is not available, it is still possible to generate reliable pharmacophore models, provided that at least one or more molecules that bind to the target receptor of interest are known. Within this so-called ligand-based approach, conformers of the active ligands are superimposed to identify common, spatially similarly arranged structural features whose sum then constitutes the corresponding 3D pharmacophore model. However, for many biological targets, apo-protein structures or homology models are available; this is evident as over 41,000 of 150,000 (27%) X-ray structures in the Protein Data Bank (PDB)^10^ do not contain a small-molecule ligand (excluding inorganic ions and water).^11^ Moreover, for many allosteric pockets, potent binders have not yet been discovered which then also do not permit the application of ligand-based modeling approaches. The most commonly used approach to enrich compound libraries for apo-sites is molecular docking.^12^ However, docking a large library of molecules is computationally quite intense and often requires simplifications to be feasible, for example, docking only pre-generated rigid molecule conformations.^6,13,14^ While it is possible to generate pharmacophore features solely from an apo-crystal structure or from a homology model, the derivation of high-quality apo-site pharmacophores is generally a challenging task because there may be a large number of potential interactions with the binding site residues—a criterion where no single molecule is likely to fulfil.^15^ Recently, our group has developed a grid-based method for molecular interaction analysis that enables the identification and visualization of potential binding site interaction hot spots.^16^ In addition, several other methods exist to predict pharmacophoric interactions; most of these similar grid-based approaches work by calculating interaction energies of distinct probes for certain feature types located at the points of a three-dimensional (3D) lattice superimposed with a biomolecule.^14−17^ Yet, due to the near infinite number of combinations of possible pharmacophore features, it is difficult to create a proper query pharmacophore for VS runs.^15^ Pharmacophoric features may be filtered by buriedness or predicted interaction energies but are still very reliant on subjective decisions to obtain a pharmacophore model that is reasonable for VS, especially when no other information apart from the 3D structure of the apo-site is available.^15^

In the last two decades, several methods for receptor-focused pharmacophore generation have been developed in an attempt to fill this gap in the list of pharmacophore modeling approaches.^15,17−23^ However, none of these methods were established as a gold standard (see a recent publication by Volkamer et al. for an excellent overview).^17^ This is mostly due to the qualitative or conceptional nature of those methods, or the lack of reliant scoring to limit the number of pharmacophore features. In need of a method to reliably generate apo-site pharmacophores for several projects, we sought to develop a streamlined workflow, called *apo2ph4*, for rapid and straightforward receptor-based pharmacophore generation. The approach presented herein utilizes fragment docking to generate an array of SB pharmacophore models, which are then clustered and scored based on feature density to output pharmacophore models suitable for VS. Despite being prototypical in nature, *apo2ph4* has shown tremendous prospective value for in-house projects (unpublished work); hence, we have decided to make it publicly available. A short qualitative evaluation as well as a prospective use case for discovering novel GABA_A_R ligands demonstrating the workflow’s prospective value will be presented herein. In order to probe whether *apo2ph4* can be successfully applied to a wider variety of systems, the workflow was employed to all 15 targets of the recently published LIT-PCBA dataset.^24^ Also here, *apo2ph4* has shown excellent performance, and the obtained results will be presented and discussed in this work.

## Methods

**Figure 1:**
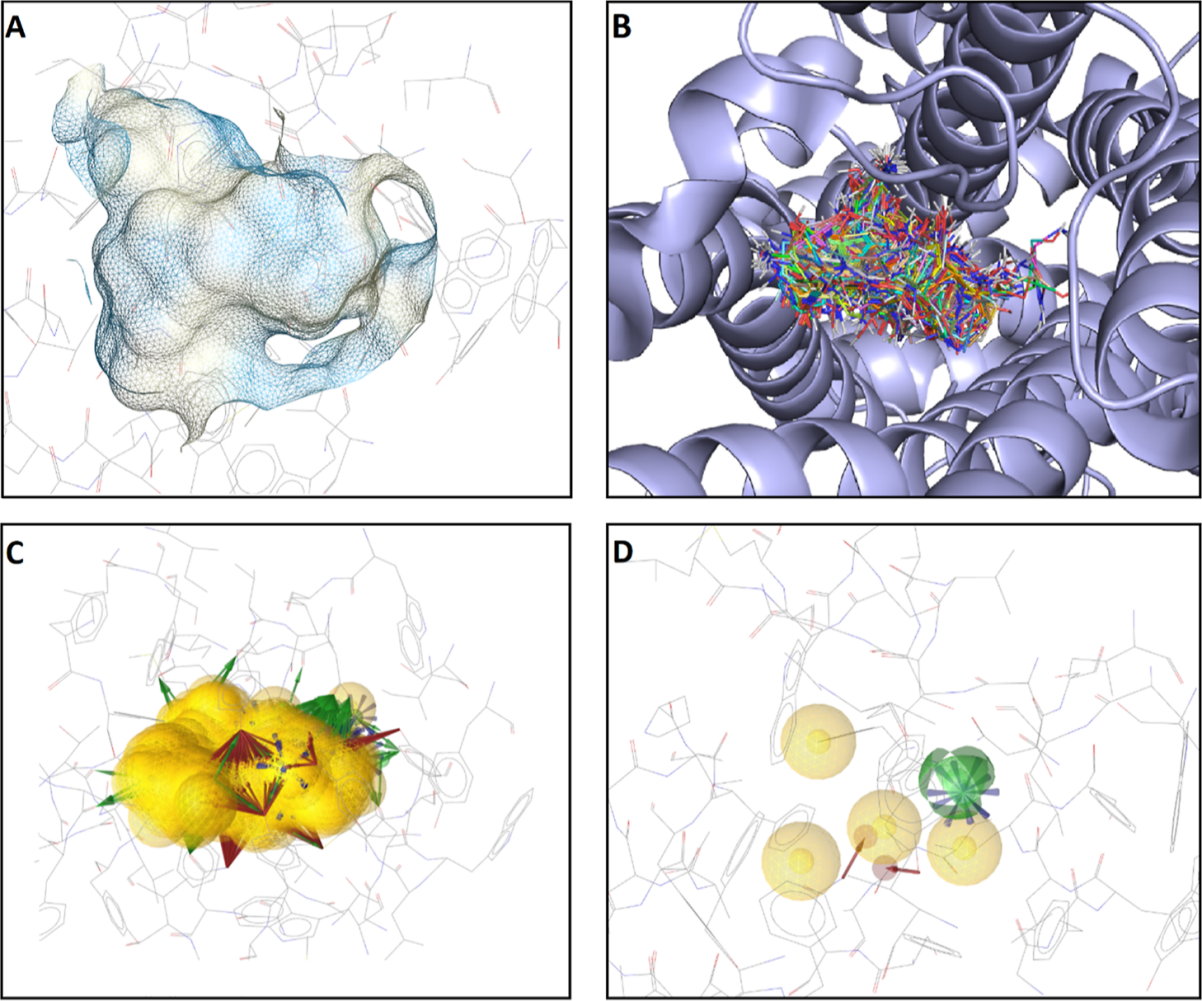
Overview of the separate steps performed by the *apo2ph4* workflow: (A) selection of a suitable binding site. (B) Docking of a diverse set of medium-sized fragments into this binding site. (C) Generation of a SB pharmacophore model for each binding pose of each docked fragment. (D) Scoring, clustering, and filtering of features to output a single pharmacophore model ready for VS.

The *apo2ph4* workflow comprises four steps: (A) selection of a (potential) binding site of a resolved protein structure or a homology model, (B) docking of a fragment library into that binding site, (C) generation of SB pharmacophore models for each selected docking pose, and (D) scoring, clustering, and filtering of features to output a single pharmacophore model ready for VS (Figure 1). The necessary scripts for the *apo2ph4* workflow are available on GitHub.^25^

If a ligand occupying the protein structure is used as a center point for docking, its center of mass is calculated. If other molecules are present in the protein structure, they must be removed beforehand. For apo-proteins, or in an effort to generate a pharmacophore for a potential allosteric binding pocket, center coordinates may either be defined manually or by previously placing dummy atoms in the desired binding site which can be conveniently done by using PyMol.^26^ If structurally important water molecules or non-metal containing co-factors are contained within the protein structure file, a PDB file must be prepared manually for docking and subsequent steps.

### Fragment Library

We have chosen a subset of Prestwick Chemical’s fragment set for use as a fragment library.^27^ This library contains a diverse set of 1456 lead-like fragments which have been derived from FDA approved drugs and was originally intended for fragment-based in vitro testing. To limit the time required for docking, a subset of 200 molecules was picked using the RDKit^28^ diversity KNIME^29^ node using a random seed. The molecular weight of the fragments ranges from 81 to 301 Da following a roughly Gaussian contribution. This is also the case for other critical descriptors such as *c* Log *P* and hydrogen donor and acceptor counts. As required for later steps, the number of potential ligand-based pharmacophore features is also calculated at this point using CDPKit.^30^

### Docking

The protein and the fragments are then converted into the PDBQT format, which is required for docking with AutoDock Vina^31^ via a shell script employing MGLTools 1.5.6 (included in AutoDockTools4^32^). This conversion may alternatively be performed by using OpenBabel.^33^ The PDBQT files are then submitted to docking using AutoDock Vina. A 20 × 20 × 20 Å grid box was chosen, and exhaustiveness was set to 20—it should be noted that using the default value of 8 is also acceptable, which may result in a slightly attenuated pharmacophore model quality, to significantly reduce the time required for docking if needed. Only the best two poses per fragment are kept if they are within an energy threshold of 2 kcal/mol. Additionally, AutoDock4^32^ grid energies, using a 30 × 30 × 30 Å box with 0.3 Å spacing, of the protein complex were calculated using AutoGrid as they are needed for later steps.

### Generation of SB Pharmacophore Models for Each Fragment

Using a python script employing the PyMOL API, mock co-crystal complexes of docking poses of each docked fragment with the protein structure were generated and saved as PDB files. Utilizing the batch version of KNIME, these files were then submitted to LigandScout’s SB pharmacophore-creator KNIME node and the generated pharmacophores were saved in a single LigandScout PML file.

### SB Pharmacophore Generation for Fragments

By means of a python script, pharmacophore features were then extracted from the PML file using a functionality provided by CDPKit^30^ and got binned according to the feature type [hydrophobic (H), aromatic (AR), positively ionizable (PI), negatively ionizable (NI), hydrogen bond donor (HBD), hydrogen bond acceptor (HBA), and exclusion volume]. In the next step, distances between all features of the same type are calculated and each feature is assigned a score that depends on the density (number and distance) of neighboring features (eq 1). For every neighboring feature, a value is added to the total score, strongly rewarding features within close proximity. For this, a relatively steep logistic scoring function is applied: features that share the same coordinates get assigned a value of nearly 1, while, for example, features with a distance (eq 1: dist) of 1 Å contribute a value of 0.5 and features with a distance of 2 Å contribute a near-zero value (0.02). The list of features is then sorted by the assigned score and pruned by discarding all features that are within 3 Å of a higher-scored feature of the same type. 3 Å was chosen as a threshold, as this is the default minimum distance two LigandScout pharmacophore features can be apart from each other without overlapping. To ensure a fairer comparison of scores between feature types, each score is normalized dividing by the number of maximum possible ligand-based pharmacophore features (eq 1: *n*_feat._) calculated earlier to yield a final score (. While this approach worked well for hydrophobic features, additional considerations had to be made for other feature types. During preliminary optimization, we discovered that PI and NI group features were often poorly placed, resulting in the same or less enrichment compared to random selection. This may be explained as very polar moieties are simply placed in a position that results in the least penalty by the docking algorithm’s scoring function. This issue could rapidly be resolved by considering the electrostatic potential map calculated via AutoGrid: PI features displaying a value over −0.7 kcal/mol and NI features displaying a value under 0.9 kcal/mol were removed after the scoring but before the pruning step.
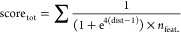
1

Unlike hydrophobic and ionizable features, aromatic features are modeled as planes because their interaction with side-chain aromatic groups must be within a certain angle to be of favorable energy. Hence, aromatic features with vastly different plane orientations should not be considered to be a similar feature for purposes of scoring. Employing DBSCAN implemented in scikit-learn,^34^ aromatic features were clustered by their plane angles and scored individually.

HBD and acceptor features may be presented as spherical features; however, as the correct angular geometry and distance between a donor and acceptor pair are critical for interactions to be energetically favorable,^35^ we opted to represent donor/acceptor features as vector features for VS.^8^ Hence, acceptor features were clustered by their initial point and donor features by their terminal point before scoring. In the default implementation, it is not possible for two vector features of the same type to be present at the same location as it is very difficult to satisfy two hydrogen bonds with the same functional group adequately. By default, if two or more vector features overlap, they are instead outputted as a spherical feature.

Exclusion volumes were treated differently as they are solely dependent on protein geometry; hence, all generated exclusion volumes were kept, with only duplicates being removed for screening efficiency. After overlapping features were removed and all scores are assigned, the defined number of features is selected based on the previously obtained score and written in LigandScout’s PML format. For most systems, it has proven to be beneficial to limit the number of features of a certain type; thus, hydrophobic features are limited to a maximum of four, PI and NI features to a maximum of one (each) and HBDs to a maximum of two, using default settings.

### Validation of *apo2ph4* by Pharmacophore Generation and Virtual Screening for Targets of the LIT-PCBA Dataset

All active and decoy datasets were used in the form provided by the LIT-PCBA website.^24,36^ By means of PyMol, the provided .mol2 files of ligands and targets were merged into a corresponding PDB file to apply *apo2ph4*. Conformations of active and inactive compounds were generated using CDPKit’s CONFORT conformer generator (confgen) using default settings, allowing up to 25 conformations per molecule to be generated.^30,37^ For VS, the generated multi-conformer SD files were then converted to LigandScout’s .ldb format via KNIME nodes provided by the LigandScout KNIME extension.^38^ To generate pharmacophore models for VS, *apo2ph4* was run using all-default settings. The number of features to be matched was determined by screening the active dataset: the highest number of features was chosen that was able to retrieve at least one active compound. For enrichment calculations using the corresponding active and decoy datasets, VS runs were carried out on a HPC cluster using LigandScout Remote.^39^ All query pharmacophore models employed in the VS runs are provided as Supporting Information.

### Pharmacophore Generation and Virtual Screening for the GABA_A_ Receptor

Using a cryo-EM Structure of the GABA_A_ receptor in complex with etomidate, we have employed the workflow presented herein, using etomidate enclosed by the C and D chains (PDB chain ID) as the center point for docking. We have generated a pharmacophore model containing seven features as a query for subsequent VS. We opted to use MolPort’s compound library (downloaded March 2020) as a VS database, which at the time of VS contained roughly 7 million commercially available compounds.^40^ Using Quacpac,^41^ compounds were set to their most favorable ionization state at the physiological pH of 7.4 and 25 conformations per compound were generated with Omega.^42^ Using LigandScout, Remote^39^ VS was performed, while allowing one feature to be omitted, yielding about 19,573 hits. As a first filtering step, only the hits with a pharmacophore fit score within the 70th percentile were kept (5872 hits remaining). Using LigandScout, the MMFF94^43^ energy of the molecules was then minimized in the protein structure. Standard properties and interacting feature counts were then calculated using LigandScout for the remaining molecules to allow a filtering according to Lipinski’s Ro5.^44,45^ Additionally, molecules having more than 10 rotatable bonds or a polar surface area greater than 140 Å^2^ were filtered out (Veber’s Rule).^46^ In order to comply with the known high lipophilicity of the pocket, only hits displaying a *c* Log *P* of greater than 2 were kept (4273 hits remaining). To filter out molecules with severely shifted poses due to minimization, their RMSD from the original poses was calculated and only molecules within an RMSD below 2 Å were kept (2925 hits remaining). Molecules which were strongly deformed as a result of minimalization (e.g., out of plane aromatic rings) were filtered out by only keeping molecules displaying a negative MMFF94 binding enthalpy, which was calculated within LigandScout (608 hits remaining). The remaining molecules were then redocked into the binding pocket using AutoDock Vina (default settings), and only molecules were kept if at least one docking pose resembled the pose minimized earlier within an rmsd of 1.5 Å. Following this rather harsh filtering procedure, 420 hits remained, which were sorted by their pharmacophore fit score. After visual inspection, comparing their pharmacophore fit with the query pharmacophore, consideration of actual commercial availability and diversity, a subset of 20 hits was chosen for subsequent in vitro assays.

### Animals and Animal Welfare

All experiments involving animals were approved by the Austrian Animal Experimentation Ethics Board in compliance with the European convention for the protection of vertebrate animals used for experimental and other scientific purposes ETS no.: 123, which is in line with the EU Directive 2010/63/EU (GZ 66.011/0123-II/3b/2015 and 66.006/0029-WF/V/3b/2014). Female *Xenopus laevis* (*X. laevis*) frogs were purchased from NASCO (Fort Atkinson, USA) and kept in groups in temperature-controlled, continuous-flow water tanks (20 ± 1 °C), %; a 12 h light–dark cycle was in operation (lights on from 07.00 to 19.00).

### Ion Channel Expression in *X. laevis* Oocytes and Two-Microelectrode Voltage Clamp Assay

Preparation of stage V–VI oocytes from *X. laevis* and expression of recombinant GABA_A_ receptors (α1β2γ2s) in *X. laevis* oocytes by cRNA injection were performed as previously described.^47^ Female *X. laevis* frogs were anesthetized by 15 min incubation in a 0.2% MS-222 (methanesulfonate salt of 3-aminobenzoic acid ethyl ester; Sigma-Aldrich, Vienna, Austria) solution before removal of parts of the ovaries. Follicle membranes from isolated oocytes were enzymatically digested with 1 mg/mL collagenase (Type 1A; Sigma-Aldrich, Vienna, Austria). Selected oocytes were injected with 10–50 nL of DEPC-treated water (diethyl pyrocarbonate; Sigma, Vienna, Austria) containing different cRNAs at a concentration ranging between 200 and 3000 pg/nL/subunit. To ensure expression of the γ2S subunit, cRNAs were mixed in a ratio of 1:1:10.^48^ Oocytes were stored at +18 °C in ND96 solution (all from Sigma-Aldrich, Vienna, Austria). Two-microelectrode voltage clamp measurements were performed between days 1 and 5 after injection of cRNA of the respective subunits, using a TURBO TEC 03X amplifier (npi Electronic) at a holding potential of −70 mV and pCLAMP 10 data acquisition software (Molecular Devices). The bath solution contained 90 mM NaCl, 1 mM KCl, 1 mM MgCl_2_, 1 mM CaCl_2_, and 5 mM HEPES (pH 7.4). Electrode filling solution contained 3 M KCl. Test solutions (150 μL) were applied to the oocytes at a speed of 300 μL/s using the ScreeningTool (npi electronic) automated fast perfusion system. GABA EC_3–5_ was determined through a concentration–response experiment with GABA. Stock solution of compounds (30 mM) in DMSO was diluted with a bath solution containing GABA EC_3–5_ to obtain appropriate working solutions according to a validated protocol. Enhancement of the *I*_GABA_ was defined as (*I*_(GABA+Comp)_/*I*_GABA_) – 1, where *I*_(GABA+Comp)_ is the current response in the presence of a given compound and *I*_GABA_ is the control of the GABA-induced chloride current. The data were analyzed using Origin Software (OriginLab Corporation, USA) and are given as mean ± SEM of 3 oocytes from ≥2 batches.

## Results and Discussion

### Parameter Selection

In order to cover a big part of the drug-like feature space within a very small number of fragments, we selected the Prestwick fragment library since it is directly derived from FDA approved drugs. To cut down computational cost, we selected a diverse subset of 200 molecules. It should be noted that a larger number of fragments appears to only very slightly improve results. Hence, when computational cost is negligible or only a small number of pharmacophore models are to be generated, a larger fragment library may be used. While a 20 × 20 × 20 Å space for docking usually far exceeds the area of a binding pocket, we opted for a rather lose box to allow ligands to dock outside the desired area instead of forcing fragments to “bind” within the desired binding pocket. In some cases, when two binding pockets are very close to each other, this area may be adjusted accordingly. For the same reason, to discard suboptimal binding poses, only the top 2 poses were kept and only if they were within calculated 2 kcal/mol of each other.

We have set a default cutoff value of −0.4 kcal/mol to eliminate unfavorable hydrophobic features. However, for very lipophilic pockets, it may be beneficial to lower the default cutoff to −0.5 to −0.6 kcal/mol for improved placement of hydrophobic features. The employed empirically determined energy cutoffs for positively and negatively ionized features seemed to be generally very applicable for most systems. However, for many systems, these very polar features still might not result in energetically favorable interactions. Thus, it is generally advisable to also perform VS runs with PI/NI features marked as optional unless there is very strong indication that such features are beneficial [e.g., for muscarinic acetylcholine receptors (mAChRs)].^49^ Introducing energy cutoffs for HBDs and acceptors, as well as aromatic features, generally did not show any benefits. While HBDs are very often accurately placed, they may sometimes be generated too frequently compared to SB pharmacophore models of reference systems and during VS against known actives. Unfortunately, we were not able to find a suitable method to discard “unnecessary” HBDs. However, as many HBDs (e.g., phenols and peptides) are associated with metabolic instability and with decreased oral bioavailability,^50−52^ data may be skewed in reference datasets of known actives. We have also attempted to consider buriedness via implementing a crude 7-axis PSP score;^53^ however, this did not seem to improve the results, likely because buriedness is already indirectly taken into account by the docking algorithm. In case there is no information about known ligands available, we recommend VS with a higher number of features but with one omitted feature—which also ensures a broader diversity of the VS hits.

### Comparison to SB Pharmacophores

**Figure 2:**
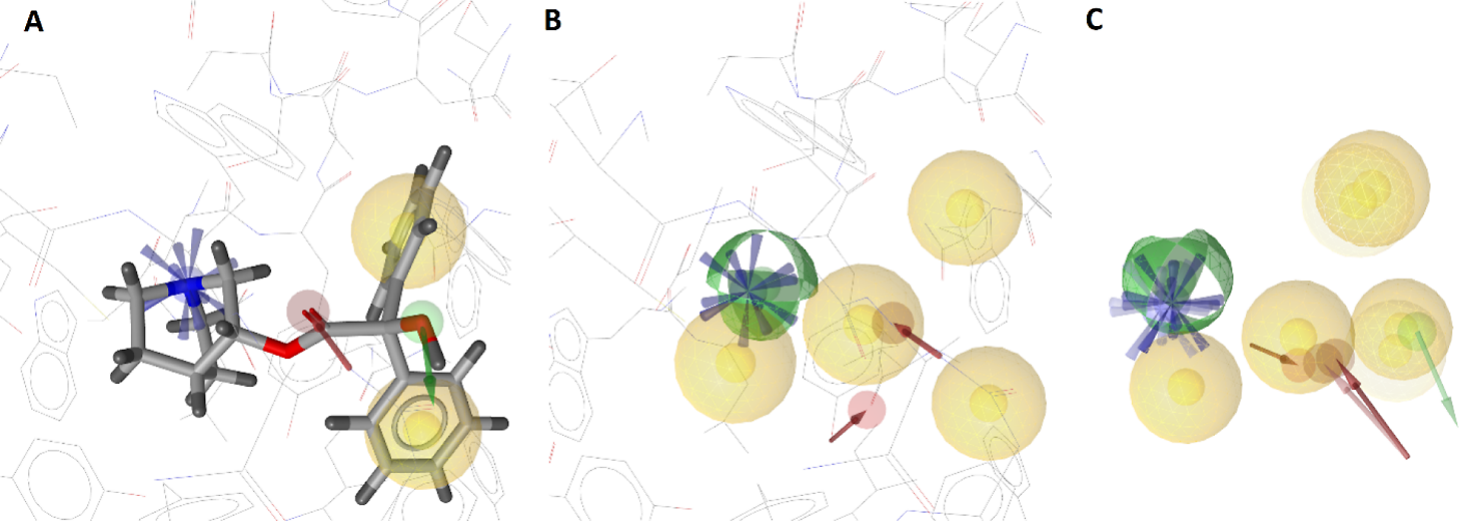
Pharmacophore models derived from PDB-entry 3UON: (A) SB pharmacophore model obtained using LigandScout. (B) Pharmacophore model obtained by *apo2ph4*. (C) Superposition of the SB (light/translucent) and the *apo2ph4* (dark) pharmacophore model. Legend: yellow sphere = hydrophobic feature; red vector = directed HBA feature; green vector = directed HBD feature; green sphere = undirected HBD feature; blue star = positive ionizable feature.

Due to our experience in the development of orthosteric antagonists targeting mAChRs,^54−56^ we decided to qualitatively evaluate the predictive value of the *apo2ph4* workflow using a crystal structure of M2 muscarinic acetylcholine receptor subtype (PDB-entry 3UON^57^). It should be noted that neither 3UON nor any other muscarinic acetylcholine receptor was used during internal optimization. After following the general procedure, an 8-feature pharmacophore model was obtained using default settings, comprising 4 hydrophobic, 2 HBA, 1 HBD, and 1 PI feature (Figure 2).

Using LigandScout, a SB pharmacophore model was created from 3UON to yield a 5-feature pharmacophore model consisting of 2 hydrophobic, 1 HBA, 1 HBD, and 1 PI feature (Figure 2). Both of the hydrophobic features from the SB model were closely matched by the ones generated without ligand information (within 0.62 and 0.66 Å). The same is true for the HBA interacting with Asn404^6.52^ and the PI feature interacting with Asp103^3.32^ (superscript numerals refer to the Ballesteros–Weinstein numbering scheme^58^ for GPCRs), which were found within 0.76 and 0.41 Å, respectively. The HBD feature of the SB pharmacophore interacting with Asn404^6.52^ was not present in the *apo2ph4* pharmacophore. However, as evident by other published crystal structures or by recently developed ligands,^55,56^ this is not an essential feature (e.g., PDB-entry 4MQS^59^). Comparing the *apo2ph4* pharmacophore with PDB-entry 5ZKB,^60^ a different crystal structure of the muscarinic receptor, the second HBA feature generated by the tool closely matches the respective HBA feature interacting with Tyr104^3.33^ from the SB model within 3.46 Å (Figure S1). The generated HBD feature interacting with Ser107^3.36^ from PDB-entry 5ZKB may also be matched with the HBD generated by the *apo2ph4* workflow within a distance of 2.85 Å. While these observed distances appear to be high, superposition of 6UON and 5ZKB reveals that the oxygen atoms of side chains of Tyr104^3.33^ and Ser107^3.36^ are shifted by distances of 2.58 and 2.91 Å, respectively, in the same direction as the respective features. While there are no crystal structures of the M2 muscarinic acetylcholine receptor directly matching the two remaining hydrophobic features generated by *apo2ph4*, several known mAChRs ligands may possibly provide this feature. For example, the SB pharmacophore of the top scored docking pose generated for antagonist Tropicamide^61^ closely matches the hydrophobic feature near Asp103^3.32^ within 0.93 Å while also matching 4 other generated features within 2 Å (Figure S2). A SB pharmacophore of the top scored docking pose of muscarinic agonist Cevimeline^62^ reveals one hydrophobic feature placed within 1.69 Å of the last not yet assigned generated feature(Figure S2). The fact that six of the eight generated features could directly be confirmed by examining crystal structures and the remaining two features have a strong possibility to match interactions by known mAChRs ligands strongly emphasizes the quality of the obtained pharmacophoric features and their usefulness for VS.

### Systematic Evaluation of *apo2ph4*’s Performance

To probe whether *apo2ph4* can be successfully applied to a variety of systems, we decided to employ the recently published LIT-PCBA dataset.^24^ Older and more established datasets such as DUD-E^63^ are commonly employed for benchmarking VS techniques; however, they are known to contain significant biases and tend to overestimate the performance of VS techniques as elaborately discussed by Rognan and co-workers.^24^ While DUD-E only provides computer-generated decoys, LIT-PCBA’s decoy datasets contain true inactive compounds that were obtained from high-confidence high-throughput screening data. In addition, several measures were taken to de-bias the datasets. Hence, *apo2ph4* was applied to PDB structures of all 15 targets of the dataset, using only default settings without any target-specific optimizations (Table 1). If only one active was retrieved during VS, possibly inflating enrichment, a second system is shown. Strikingly, VS runs using the *apo2ph4*-generated pharmacophore models led to significant enrichment for 13 out of 15 targets ranging from 1.7 to 24.2. Listed enrichment factors were calculated for the database subset retrieved as VS hits, that is, if a hit rate of 0.25% was achieved, the enrichment factor has been calculated for 0.25% of the total database (EF_0.25%_). This had to be done as most of the VS runs resulted in a hit rate of less than 1%, rendering it impossible to calculate standard metrics like EF_1%_. These results unequivocally show that *apo2ph4* is applicable to a wide range of systems and VS runs using queries generated by *apo2ph4* can be expected to lead to often significant enrichments of actives.

**Table 1:** Quantitative and Systematic Evaluation of *apo2ph4* using the LIT-PCBA Dataset

entry	target	PDB template	actives	inactives	hits (actives)	hits (inactives)	no. of features^c^	hit rate (%)	EF_hits_
**1a**	ADRB2	4LDE	17	312,483	3	8243	3	2.64	6.7
**2a**	ALDH1	5AC2	7168	137,695	2	16	6	0.01	2.2
**3a**	ESR_ago	2QR9	13	5583	2	64	4	1.18	13.0
**4a**	ESR_ant	6B0F	102	4948	1	2	4	0.06	16.5
**4b**		2POG			5	60	4	1.29	3.8
**5a**	FEN1	5FV7^a^	369	355,402	3	881	5	0.25	3.3
**6a**	GBA	3RIK	166	296,052	3	384	5	0.13	13.8
**7a**	IDH1	4I3L	39	362,049	1	382	5	0.11	24.2
**7b**		5TQH			5	8597	4	2.38	5.4
**8a**	KAT2A	5MLJ	194	348,548	1	205	5	0.06	8.7
**8b**		5MLJ			5	2741	4	0.79	3.3
**9a**	MAPK1	3W55	308	62,629	4	156	5	0.25	5.1
**10a**	MTORC1	4FAP	97	32,972	32	8933	4	27.11	1.2
**10b**		4FAP_EF1%_^b^			4	333	4	1.00	4.0
**11a**	OPRK1	6B73	24	269,816	0	12	5	0.00	0.0
**12a**	PKM2	3ME3	546	245,523	4	585	6	0.24	3.1
**13a**	PPARG	5Z5S	27	5211	1	10	5	0.19	17.6
**13b**		5Y2T			3	340	4	6.55	1.7
**14a**	TP53	4AGQ	79	4168	3	210	4	5.02	0.8
**15a**	VDR	2A2I	884	355,388	13	2917	5	0.82	1.8

a A PI feature was removed from the pharmacophore model manually because a magnesium atom is known to occupy the location the feature was placed in. Currently, *apo2ph4* cannot account for coordinating metal ions automatically.

b Due to exceptionally high hit rate, only ranked hits representing top 1% of the entire dataset were used to calculate EF.

c Number of features present in the pharmacophore model used for VS.

### Prospective Value

We originally have developed the *apo2ph4* workflow for internal purposes to find ligands acting as allosteric kinase activators, and it has led us to discover new binders for previously unknown binding pockets, which served as the start of a drug discovery campaign which is currently under development and not yet published. Similarly, we have applied the workflow to the GABA_A_ receptor, which also served as a spark in the development of novel alpha-6 selective GABA_A_R positive allosteric modulators. This publication features our third VS campaign applying the *apo2ph4* workflow. Recently, a new cryo-EM structure (PDB-entry 6 × 3 V^64^) of the human GABA_A_ receptor in complex with etomidate was released. As this is the suspected binding site of β2/3 subunit-selective GABA_A_R modulators described earlier by our group as well as valerenic acid (VA) and loreclezole, we are interested in the development of novel ligands with improved pharmacodynamics.^65^

**Figure 3:**
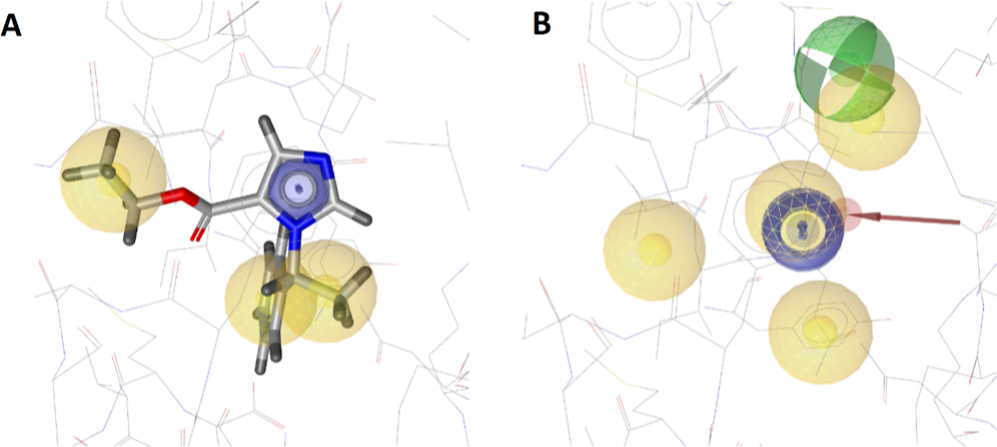
Pharmacophore models derived from PDB-entry 6 × 3 V: (A) SB pharmacophore model obtained by LigandScout. (B) Pharmacophore model obtained by *apo2ph4*. Legend: yellow sphere = hydrophobic feature; red vector = directed HBA feature; green sphere = undirected HBD feature; blue disk = aromatic feature.

**Figure 4:**
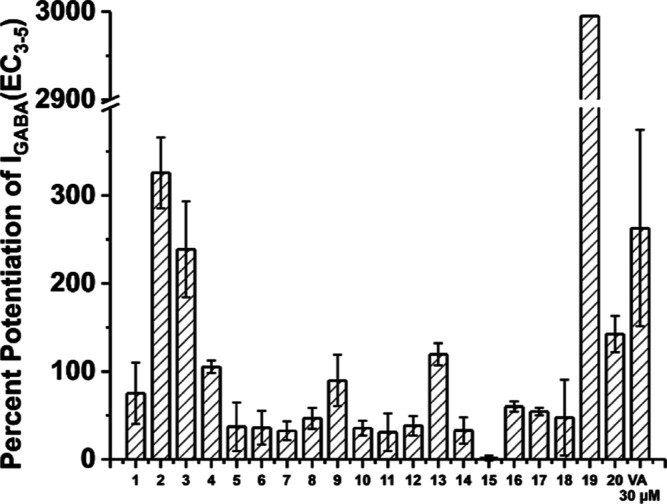
Screening results (30 μM) showing *I*_GABA_ enhancement through α1β2γ2S GABA_A_ receptors of the generated compound library of hits compared to VA; error bars for compound 19 are not shown, for this see supplementary Figure S3. The data are presented as mean values ± SEM, *n* = 3.

While in this case, a protein–ligand complex is available, upon generating a SB pharmacophore model with LigandScout, it quickly became apparent that this pharmacophore may not be suitable for VS: with three hydrophobic and one aromatic features, the number of features is not only very low but also, without any polar features, extremely generic and leads to an extremely high hit rate when used as a query pharmacophore (Figure 3). The generated seven-feature pharmacophore, in contrast, consists of 4 H, 1 AR, one HBA, and one HBD feature (Figure 3). In order to generate a more diverse set of hits, VS was performed with one omitted feature. After several filtering steps, 20 hits were purchased for in vitro testing (see Methods section for details). In vitro screening results on α1β2γ2S channels (GABA EC_3–5_) at a concentration of 30 μM revealed that 19 of 20 purchased compounds were able to significantly potentiate GABA activation. Thirteen compounds have shown a low potentiation of 30 to 100%, 5 of them were able to potentiate GABA current by 100–350%, and one ligand **19** was able to potentiate GABA current by almost 3000% (Figure 4). Ligand structures, with the exception of ligand **19**, are disclosed in Supporting Information (Table S1). This extremely high hit rate again underscores the potential prospective value of the *apo2ph4* workflow.

As demonstrated by the previous examples, the *apo2ph4* workflow often not only allows the derivation of features that may be matched by a known ligand but is also able to generate features found in other structures or find previously unknown features. While the workflow was primarily intended to be used on apo-protein structures, its potential usefulness to generate alternative pharmacophore models from existing co-crystal structures becomes apparent.

### Usage of *apo2ph4* for In Silico Neurotoxicity Prediction

**Figure 5:**
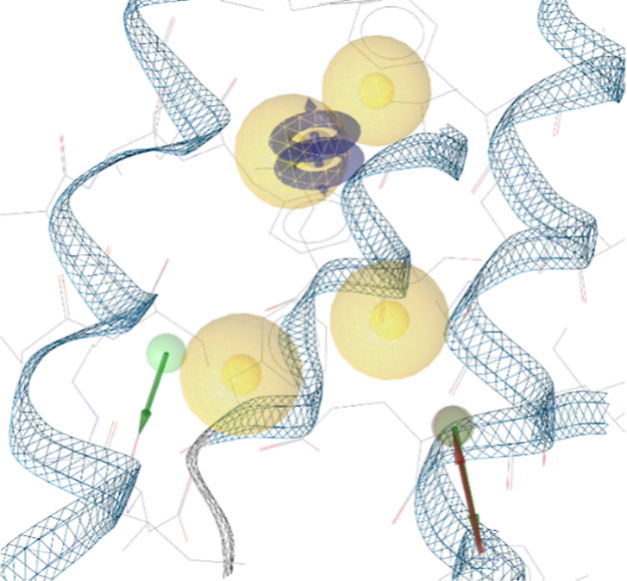
Pharmacophore model of the pregnanolone sulfate binding site of PDB-entry 6X3Z^64^ obtained by *apo2ph4*. Legend: yellow sphere = hydrophobic feature; red vector = directed HBA feature; green vector = directed HBD feature; blue disk = aromatic feature.

Clozapine is an atypical antipsychotic primarily acting antagonistic on 5HT_2A_ and dopamine receptors^66,67^ and is prone to induce seizures.^68^ This is attributed to Clozapine also binding to the GABA_A_R in an antagonistic fashion.^69,70^ Neurotoxicity, which is often caused by off-target effects, has become a huge concern for the development of new drugs.^71^ Hence, there is a big desire in being able to predict toxicity caused by off-target effects in silico. Thus, on behalf of the IM2 Project NeuroDerisk, we were developing methods to predict seizure causing off-target effects.^72^ Since the GABA_A_R is composed of five subunits of many different subtypes, a large variety of GABA_A_Rs are found in the human body, often associated with distinct physiological functions.^73^ In consequence, this means that there is a vast amount of known and possible binding sites, for many of which no ligand has been discovered yet or at least no cryo-EM structure of a ligand bound to a proposed binding site has been resolved. In addition to that, as demonstrated by the example above, SB models derived from structures of GABA_A_R in complex with a ligand often yield less than ideal pharmacophore models. Hence, we are currently applying *apo2ph4* to derive pharmacophores which may be used to discover potential off-target interactions for newly developed compounds. While we plan to publish these pharmacophores in a separate work, one pharmacophore generated for the inhibitory pregnanolone sulfate binding site^74^ of the α1β2γ2 GABA_A_R, which was derived from derived from PDB-entry 6X3Z^64^ as apo-structure, is shown here (Figure 5). To discover GABA_A_R binders with a low false negative rate, we opted to generate pharmacophore models with a larger number of features but several optional features for subsequent VS runs.

## Conclusions

In the absence of reliable pharmacophore modeling methods for apo-pharmacophore models, *apo2ph4* combines existing and commonly used tools in the field of computer-aided molecular design into a versatile workflow for generating receptor-based pharmacophore models. The combination of fragment docking and SB pharmacophore modeling seems to be a viable alternative to solely energy- or grid-based methods. This is likely as, in contrast to grid-based methods, steric and electrostatic properties of drug-like molecules are more strongly taken into account via docking rather than the use of simplified, often atomic, probes. Comparing generated pharmacophores with SB models derived from known systems, *apo2ph4* appears to be largely successful on deriving important features devoid of any ligand information. By applying *apo2ph4* to several targets of the LIT-PCBA dataset, we were able to show that *apo2ph4* is applicable to a large number of diverse systems and that the obtained pharmacophore models, on average, lead to a significant enrichment of actives in VS experiments. In addition to the examples outlined in this publication, we verified the prospective value of *apo2ph4* for two additional systems that resulted in hits currently undergoing further development. Lastly, *apo2ph4* is currently being evaluated to generate toxicophores, which will be used to detect potential off-target effects of drug candidates in silico.

## Data and Software Availability

The workflow published herein is available on GitHub.^25^ The fragment database is proprietary but may be requested from Prestwick Pharmaceuticals Inc.^27^ The MolPort compound library is proprietary and may be requested from MolPort.^40^ The 2D structures as well as the SMILES notations of the tested compounds are described in Supporting Information.

## Supporting Information Available

The described compounds in SMILES notation (TXT)
Structure and MolPort IDs of the tested compounds (PDF)
Pharmacophore models generated by *apo2ph4* for all 15 targets of the LIT-PCBA dataset (ZIP)
