# Supplementary material for: Apo2ph4: A Versatile Workflow for the Generation of Receptor-based Pharmacophore Models for Virtual Screening
Source: J Chem Inf Model. 2022 Dec 16;63(1):101–10. doi: 10.1021/acs.jcim.2c00814 (PMC9832483; doi:10.1021/acs.jcim.2c00814)
Supplement: Supplementary file 2 — ci2c00814_si_002.pdf [file ci2c00814_si_002.pdf]

Supporting Information

apo2ph4: a versatile workflow for the generation of receptor-based pharmacophore models for virtual screening

Jörg Heider, Jonas Kilian, Aleksandra Garifulina, Steffen Hering, Thierry Langer and Thomas Seidel

Additional figures

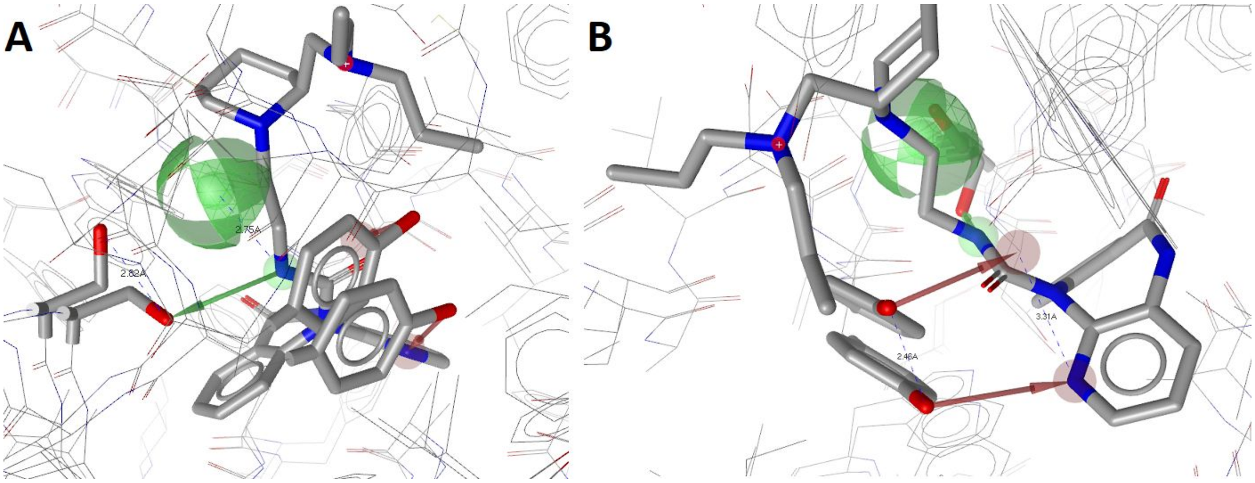

Figure S1 – Superposition of PDB-entry 3UON and 5ZKB as well as relevant HBD/HBA features of the structure-based pharmacophores obtained from PDB-entry 5ZKB using LigandScout and from PDB-entry 3UON using apo2ph4. A) The different orientation of serine in the two structures explains the distance observed between the HBD-features. B) The different orientation of tyrosine in the two structures explains the distance observed between the HBA-features.

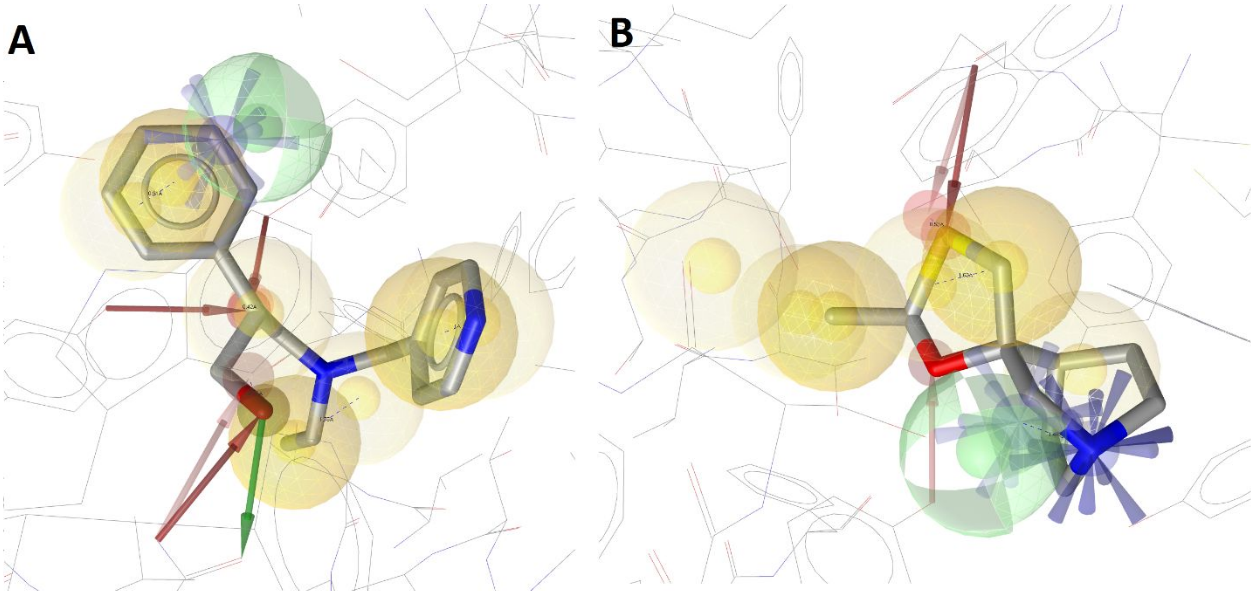

Figure S2 – Superposition of the structure-based pharmacophore of the top ranked docking pose (using LigandScout's built-in AutoDock Vina) and the pharmacophore model generated by apo2ph4: A) Docking pose of Tropicamide. B) Docking pose of Cevimeline.

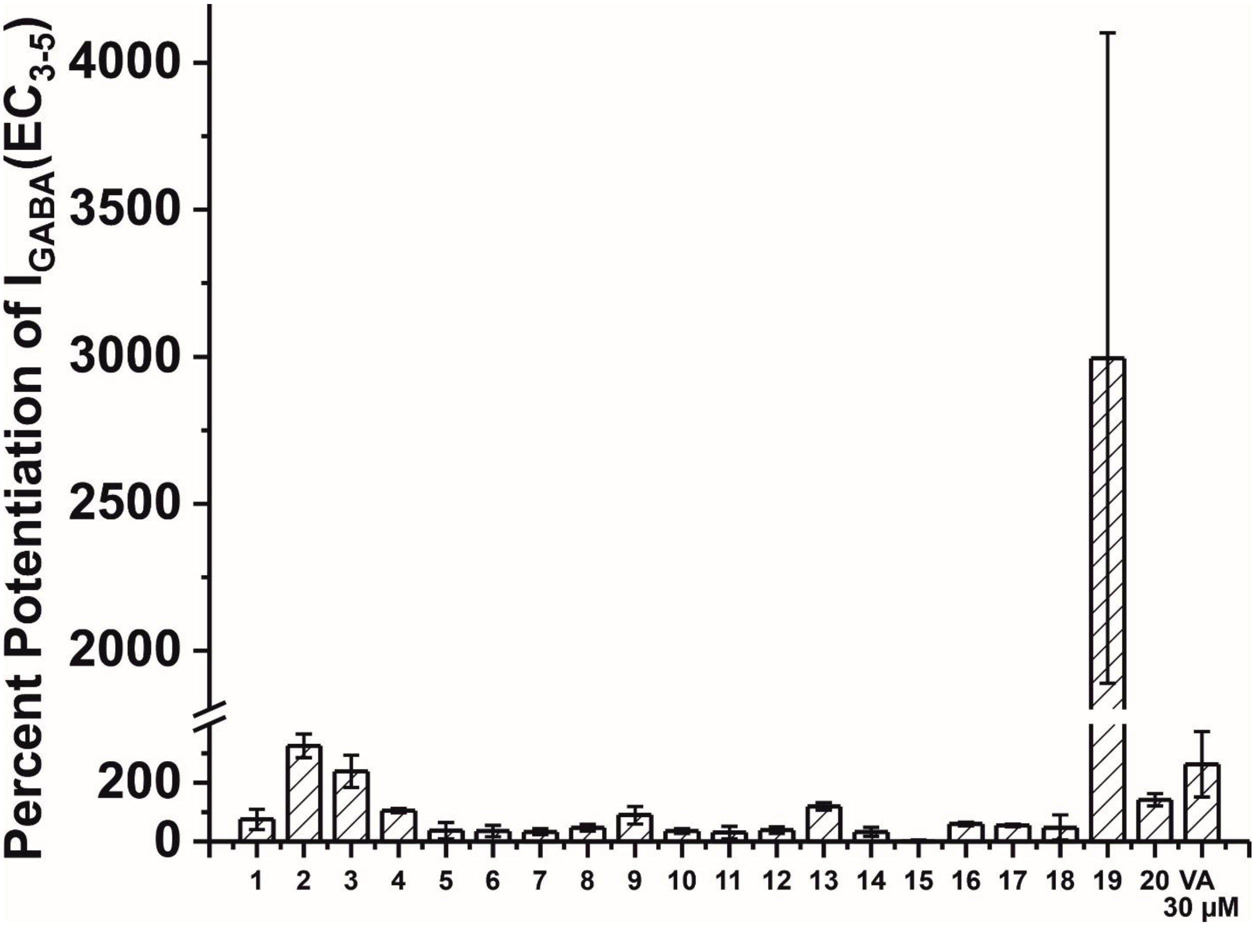

Figure S3 - Screening results (30 µM) showing I_{GABA} enhancements in α1β2γ2S $GABA_A$ receptors that could be achieved by the tested hit list compounds in comparison to VA (valerenic acid). The data are presented as mean values ±SEM, n=3.

Table S1 - 2D structure, MolPort ID and SMILES code of the tested compounds

**1**	MolPort-022-860-350	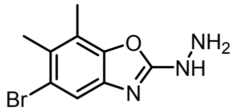	CC1=C(OC(NN)=N2)C2=CC(Br)=C1C
**2**	MolPort-028-933-565	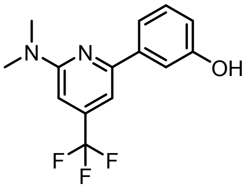	OC1=CC=CC(C2=NC(N(C)C)=CC(C(F)(F)F)=C2)=C1
**3**	MolPort-042-567-398	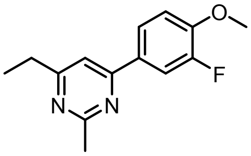	CC1=NC(C2=CC=C(OC)C(F)=C2)=CC(CC)=N1
**4**	MolPort-021-775 787	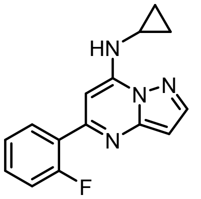	FC1=CC=CC=C1C2=NC3=CC=NN3C(NC4CC4)=C2
**5**	MolPort-045-924-819	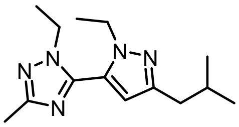	CC1=NN(CC)C(C2=CC(CC(C)C)=NN2CC)=N1
**6**	MolPort-019-800-418	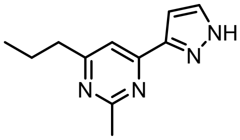	CCCC1=NC(C)=NC(C2=NNC=C2)=C1
**7**	MolPort-010-675-626	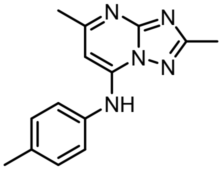	CC1=CC=C(NC2=CC(C)=NC3=NC(C)=NN23)C=C1
**8**	MolPort-002-005-699	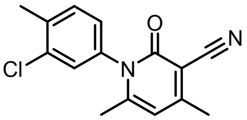	N#CC1=C(C)C=C(C)N(C2=CC=C(C)C(Cl)=C2)C1=O
**9**	MolPort-044-303-976	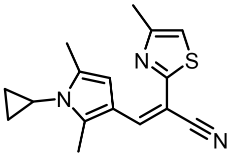	N#C/C(C1=NC(C)=CS1)=C/C2=C(C)N(C3CC3)C(C)=C2

**10**	MolPort-019-663-520	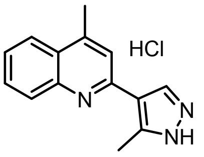	CC1=C(C2=NC3=C<br>C=CC=C3C(C)=C2)<br>C=NN1.Cl
**11**	MolPort-005-294-455	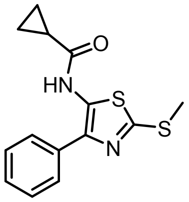	O=C(C1CC1)NC2=<br>C(C3=CC=CC=C3)<br>N=C(SC)S2
**12**	MolPort-046-065-562	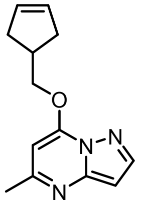	CC1=NC2=CC=NN2<br>C(OCC3CC=CC3)=<br>C1
**13**	MolPort-044-755-147	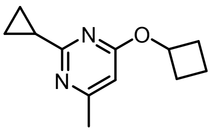	CC1=CC(OC2CCC2<br>)=NC(C3CC3)=N1
**14**	MolPort-002-907-874	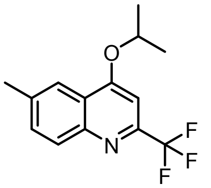	FC(C1=NC2=CC=C(<br>C)C=C2C(OC(C)C)=<br>C1)(F)F
**15**	MolPort-046-155-503	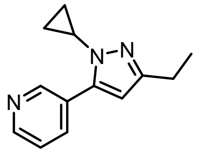	CCC1=NN(C2CC2)<br>C(C3=CC=CN=C3)=<br>C1
**16**	MolPort-004-971-957	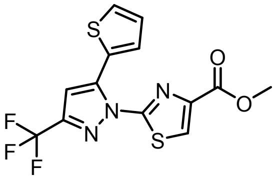	O=C(C1=CSC(N2N<br>=C(C(F)(F)F)C=C2C<br>3=CC=CS3)=N1)OC
**17**	MolPort-002-774-342	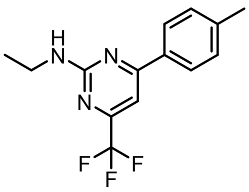	FC(C1=CC(C2=CC=<br>C(C)C=C2)=NC(NC<br>C)=N1)(F)F

**18**	MolPort-015-155-657	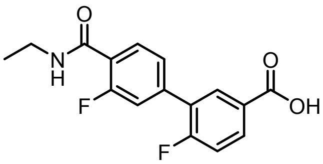	O=C(C1=CC(C2=CC=C(C(NCC)=O)C(F)=C2)=C(F)C=C1)O
**20**	MolPort-028-821-893	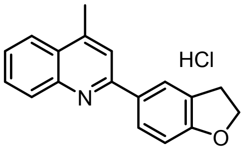	CC1=CC(C2=CC3=C(OCC3)C=C2)=NC4=CC=CC=C14.Cl
